# Prevalence of Smartphone Addiction and Its Association With Sleep Quality Among Medical Undergraduates: A Cross-Sectional Study From Eastern India

**DOI:** 10.7759/cureus.94911

**Published:** 2025-10-19

**Authors:** Geetika Singh, Saurav Singh

**Affiliations:** 1 Community Medicine, Faculty of Medicine and Health Sciences, Shree Guru Gobind Singh Tricentenary University, Gurugram, IND; 2 Pathology, Kasturba Medical College (KMC) and Hospital, Maharajganj, IND

**Keywords:** medical students, sleep quality, smartphone, smartphone addiction, undergraduates

## Abstract

Background: Smartphones have become indispensable tools, powered in part by artificial intelligence. Medical students are often overburdened and sleep-deprived due to their curricular demands, which is further aggravated by irrational and problematic smartphone usage, adversely affecting their work proficiency and health. Therefore, this study aimed to evaluate the association of smartphone addiction with the quality of sleep among medical students, along with other background variables like gender, age, year of study, etc.

Materials and methods: A cross-sectional study was conducted among 314 medical students from Netaji Subhas Medical College and Hospital, Patna, using an online self-administered questionnaire consisting of three parts: sociodemographic characteristics, Smartphone Addiction Scale (SAS-SV), and Pittsburgh Sleep Quality Index (PSQI). The Pearson correlation coefficient was used to correlate SAS scores and PQSI scores.

Results: The mean SAS-SV score was 34.01 + 9.78. The prevalence of smartphone addiction was found in 46.2% of females and 57.5% of males. The mean PSQI global score was 7.28 + 3.86. A majority, 209 (66.6%), of students had poor sleep quality as assessed by the PQSI scale. There was a positive correlation between overall PQSI scores and SAS scores (r=0.172, p<0.01). Significant association was also found between the place of residence (p < 0.05) and SAS scores (p < 0.001) with poor sleep quality, respectively.

Conclusion: Excessive smartphone use was found to be highly prevalent among medical students and significantly associated with poor sleep quality. The findings emphasize the need for awareness and behavioral interventions to promote responsible smartphone usage and improve sleep hygiene among medical undergraduates.

## Introduction

Smartphones, as widely used technological tools, have become an integral aspect of modern life owing to their various advantages, including quick access to information, enhanced social interaction, utility in professional settings, portability, convenience, and compact design [1]. As per the Statista survey report (2025), India had approximately 700.58 million smartphone users in 2024, making it the country with the second-largest number of smartphone users globally, following China [2]. In recent times, there has been a remarkable increase in smartphone usage for online access, even among young adults aged 18 to 29 years, with nearly 22% reporting that they check their phones at frequent intervals, often every few minutes. This marked rise in smartphone usage has raised concerns regarding its overuse and potential for addiction, particularly among students, often resulting in adverse psychological, social, and physical health outcomes such as neck pain, accidents, depression, and sleep disturbances [3,4].

Various theoretical frameworks have been put forward to elucidate how excessive use of electronic media devices, such as smartphones, leads to sleep disturbances. These include heightened psychophysiological arousal, disruption of circadian rhythms due to exposure to bright light, effects of electromagnetic radiation, and physical discomfort resulting from extended usage. Furthermore, both lack of sleep and a poor sleep pattern can be associated with a craving for high-calorie foods, a greater likelihood of alcohol abuse, self-harm, suicidal tendencies, excessive internet use, and smoking and drinking, particularly at night. The Diagnostic and Statistical Manual of Mental Disorders, Fifth Edition (DSM-5), categorizes gambling addiction under substance-related and addictive disorders, highlighting behavioral similarities with conditions like smartphone addiction. Research indicates that smartphone addiction shares key characteristics with these disorders, namely, compulsive behavior, functional impairment, withdrawal symptoms, and tolerance. Consequently, psychologists have defined excessive and irrational smartphone use as a form of behavioral addiction, which is now emerging as one of the most widespread forms of addiction [5-7].

Medical students, already burdened with academic stress and existing sleep issues, may experience worsened mental health and increased stress due to excessive smartphone use. Numerous studies have reported that sleep disturbances and lack of adequate rest may negatively impact academic performance and cause excessive daytime sleepiness among college students [8,9]. However, there is a paucity of research conducted among medical students, particularly in this region, regarding smartphone addiction and its potential physical and psychological consequences. Therefore, this study was conducted to estimate the prevalence of smartphone addiction and evaluate its association with the quality of sleep among undergraduates of Netaji Subhas Medical College and Hospital, a medical college in Patna in Bihar, India.

## Materials and methods

This was a cross-sectional study conducted over a period of two months at Netaji Subhas Medical College, Patna, in Bihar, India, from March to April 2024.

The study population included all the students from the first to the final year of MBBS enrolled in the undergraduate medical course at the institute. The study included those students who were willing to participate. Those students having any pre-existing psychological illness or sleep disorders, and those who were on any kind of medication related to sleep or mental health issues, were excluded from the study.

Data were collected using an online structured self-administered questionnaire (Appendix A) in the form of a Google Form (Google Inc., Mountain View, CA), which was circulated in the WhatsApp (Meta Platforms, Inc., Menlo Park, CA) group of students. The questionnaire covered sociodemographic details (age, gender, residence, academic year), the Smartphone Addiction Scale (SAS-SV) [10], and the Pittsburgh Sleep Quality Index (PSQI) [11].

The SAS-SV, a 10-item tool developed in South Korea and validated in English, was utilized in this study. It assesses five key domains: daily-life disturbance, withdrawal symptoms, virtual relationships, overuse, and tolerance. For each item, participants expressed their opinion on a six-point scale ranging from 1 (strongly disagree) to 6 (strongly agree). The scale specifies different cut-off scores for males and females, with scores above 31 for males and above 33 for females indicating smartphone addiction. Higher scores reflect greater severity of addiction. The SAS-SV is considered a valid and reliable instrument, with a reported Cronbach’s alpha of 0.967 [10].

Sleep quality was evaluated using the PSQI among medical students. The PSQI included 19 self-rated items that produced seven component scores: subjective sleep quality, sleep latency, sleep duration, habitual sleep efficiency, sleep disturbances, use of sleeping medication, and daytime dysfunction. Each component was scored from 0 to 3, resulting in a global score ranging from 0 to 21, with higher scores reflecting poorer sleep quality. Participants with a global PSQI score of 5 or less were categorized as 'good sleepers,' while those scoring above 5 were considered 'poor sleepers.' The PSQI has good internal consistency and reliability, with a Cronbach’s alpha of 0.83 [11].

Statistical analysis

Data were collected and entered in Microsoft Excel (Microsoft Corp., Redmond, WA); it was doubly checked for any errors, and data were analyzed using IBM SPSS Statistics software, version 26.0 (IBM Corp., Armonk, NY). Categorical data were summarized as proportions/percentages, and numerical data were summarized as mean and standard deviation. The chi-square test was used to compare sleep quality among participants based on demographic characteristics. The Pearson correlation coefficient was employed to assess the relationship between SAS-SV scores and PSQI scores.

Ethical considerations

Prior to the start of the study, approval of the Clinical Research Ethics Committee of Netaji Subhas Medical College was obtained via letter no. CREC/2024/11 dated 23/02/2024.

## Results

Sociodemographic profile

A total of 314 students participated in the study. The mean age of students was 20.64 + 1.98 years. There were 221 (70.4%) male and 93 (29.6%) female students. The majority (222, 70.7%) of students resided in the hostel, while 51 (16.2%) lived at home with their families. Ninety-nine (31.5%) students were in the first year, 96 (30.6%) in the second year, and 73 (23.2%) in the third year, while 46 (14.6%) were studying in the final professional year.

Smartphone usage

The average screen time spent on smartphones was more than two hours for most, i.e., 223 (71.0%) of students. The overall mean SAS-SV score was 34.01 + 9.78. More than half of the 170 students (54.1%) were found to have smartphone addiction as per the SAS-SV.

Table 1 describes the responses of the students based on the Likert scale using the SAS-SV questionnaire. Half of the students, 162 (51.6%), agreed that they missed planned work due to smartphone use. Around 120 (38.2%) students agreed they were ‘having a hard time concentrating in class, doing assignments, etc.,’ while 135 (43%) felt pain in the wrists/back of neck. Around 113 (36%) agreed to ‘will not be able to stand not having a smartphone’ and ‘feeling impatient & fretful when I am not holding my smartphone.’ A high percentage of participants (211, 67.2%) agreed to ‘having my smartphone in my mind even when I am not using it.’ About 122 (40%) of students believed that ‘I will never give up using a smartphone even when my daily life is already greatly affected.’ Most, i.e., 211 (67.2%), of students agreed to ‘constantly checking my smartphone so as not to miss conversations between other people on Twitter or Facebook,’ while 253 (80.5%) admitted to using smartphones longer than intended. More than half (190, 60.5%) agreed that ‘the people around me tell me that I use my smartphone too much.’

**Table 1:** Responses based on the Likert scale using the Smartphone Addiction Scale-Short Version (SAS-SV) questionnaire

S.No	Question	Strongly Disagree (1)	Disagree (2)	Weakly Disagree (3)	Weakly Agree (4)	Agree (5)	Strongly Agree (6)
1.	Missing planned work due to smartphone use	49(15.6)	79(25.2)	24(7.6)	42(13.4)	86(27.4)	34(10.8)
2.	Having a hard time concentrating in class, doing assignments, etc.	44(14.0)	101(32.2)	49(15.6)	51(16.2)	44(14.0)	25(8.0)
3.	Feeling pain in the wrists/back of the neck	44(14.0)	91(29.0)	44(14.0)	76(24.2)	45(14.3)	14(4.5)
4.	Will not be able to stand not having a smartphone	36(11.5)	107(34.1)	58(18.5)	58(18.5)	42(13.4)	13(4.1)
5.	Feeling impatient & fretful when I am not holding my smartphone	47(15.0)	97(30.9)	57(18.2)	54(17.2)	52(16.6)	7(2.2)
6.	Having my smartphone in my mind even when I am not using it	31(9.9)	40(12.7)	32(10.2)	115(38.6)	79(25.2)	17(5.4)
7.	I will never give up using a smartphone, even when my daily life is already greatly affected	40(12.7)	97(30.9)	55(17.5)	74(23.6)	35(11.1)	13(4.1)
8.	Constantly checking my smartphone so as not to miss conversations between other people on Twitter or Facebook	32(10.2)	44(14.0)	27(8.6)	78(24.8)	79(25.2)	54(17.2)
9.	Using my smartphone longer than I had intended	16(5.1)	27(8.6)	18(5.7)	105(33.4)	103(32.8)	45(14.3)
10.	The people around me tell me that I use my smartphone too much	36(11.5)	55(17.5)	33(10.5)	105(33.4)	61(19.4)	24(7.6)

**Figure 1:**
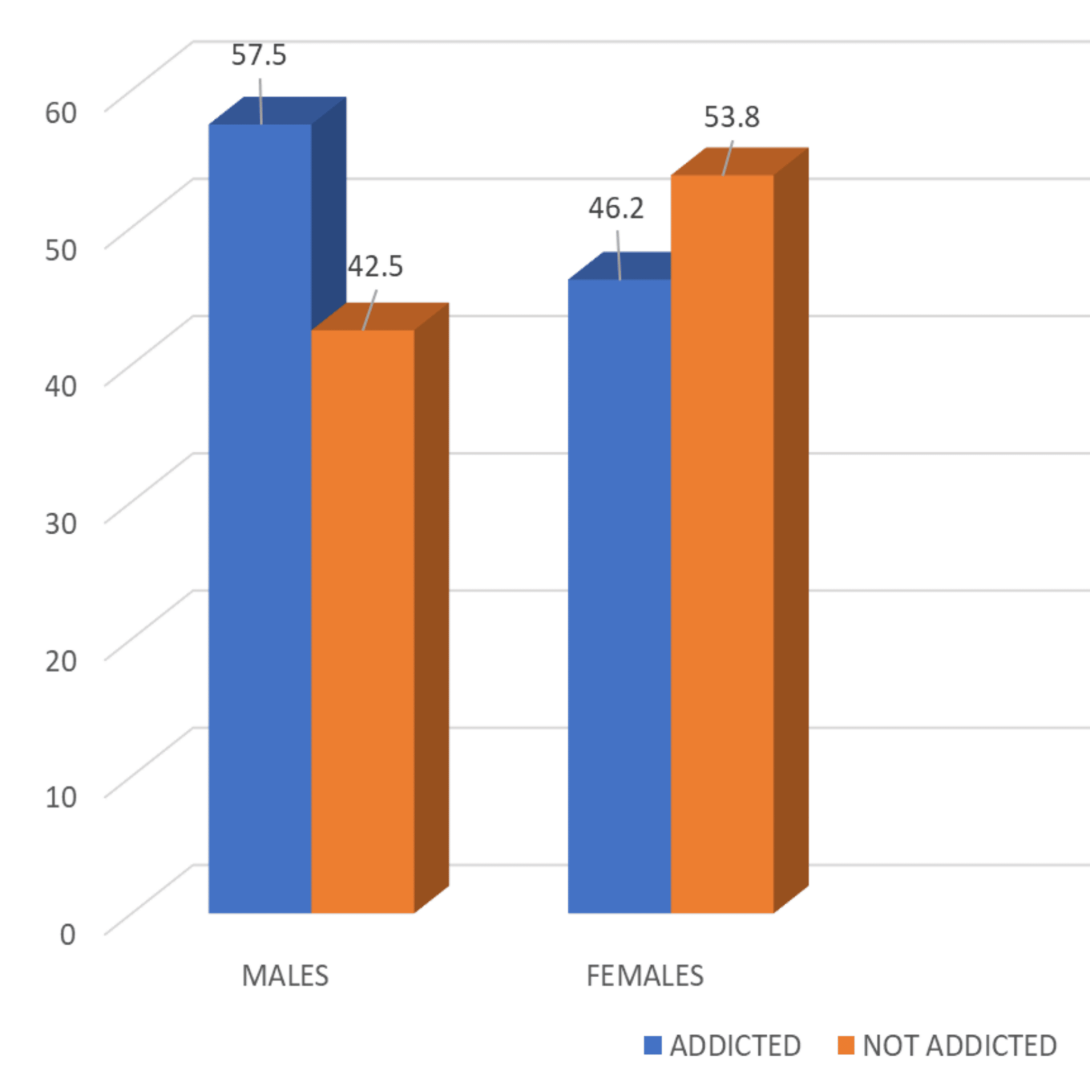
Gender-wise prevalence of smartphone addiction (%)

Regarding the gender-wise prevalence of smartphone addiction, 127 (57.5%) male students were found to be addicted as compared to 43 (46.2%) females, and this was found to be slightly statistically significant (p=0.045) (Figure 1).

Sleep quality

The majority (209, 66.6%) of students were poor sleepers, having PSQI scores more than 5, while the remaining 105 (33.4%) students had better sleep quality (PSQI scores < 5) (Table 2).

**Table 2:** Distribution of the students based on Pittsburgh Sleep Quality Index (PSQI) scores (N=314)

Sleep Quality	Number (%)
Good sleep quality (PSQI score ≤5)	105 (33.4%)
Poor sleep quality (PSQI score >5)	209 (66.6%)

Table 3 shows that the overall mean global sleep quality score among medical students was 7.28 (SD = 3.86). The components with the highest mean scores, indicating the poorest aspects of sleep quality, were sleep efficiency (mean = 2.26, SD = 1.86) and sleep latency (mean = 1.24, SD = 0.84). In contrast, the lowest mean scores were observed in the use of sleep medication (mean = 0.22, SD = 0.66) and subjective sleep quality (mean = 0.59, SD = 0.73).

**Table 3:** Mean scores of the students based on the Pittsburgh Sleep Quality Index (PSQI) components

Components of PSQI	Mean+ SD
Subjective sleep quality	0.59 + 0.73
Sleep latency	1.24 + 0.84
Sleep duration	1.18 + 0.79
Sleep efficiency	2.26 + 1.86
Sleep disturbance	1.10 + 0.64
Use of sleep medication	0.22 + 0.66
Daytime dysfunction	0.60 + 0.76
Global PSQI Score	7.28 + 3.86

Poor sleep was reported by 143 males (64.7%) and 66 females (71%). This difference observed was statistically insignificant. No statistically significant association was found between age groups or year of study and sleep quality. The students living in the hostel had poorer sleep (159, 71.6%) as compared to those living outside the college campus (24, 58.5%) and those residing at their homes (26, 51.0%), and this was found to be statistically significant (p=0.01). Further, it was revealed that out of 170 students who had smartphone addiction, the majority, i.e., 135 (79.4%) students, had poorer sleep, and this was observed to be statistically highly significant. (p<0.001) (Table 4).

**Table 4:** Sociodemographic distribution of students based on sleep quality (n = 314) *P < 0.05: statistically significant; **P < 0.001: highly significant

Variables	Sleep Quality N (%)		Chi-square (df)	P-value
	Good	Poor		
Gender			1.153 (1)	0.173
Male	78 (35.3%)	143 (64.7%)		
Female	27 (29.0%)	66 (71.0%)		
Age (in years)			0.290 (2)	0.865
18-20	57 (34.5%)	108 (65.5%)		
21-23	40 (32.8%)	82 (67.2%)		
> 24	8 (29.6%)	19 (70.4%)		
Year of study			1.700 (3)	0.637
I	31 (31.3%)	68 (68.7%)		
II	29 (30.2%)	67 (69.8%)		
III	28 (38.4%)	45 (61.6%)		
IV	17 (37.0%)	29 (63.0%)		
Present address			9.303 (2)	0.01^*^
Home	25 (49.0%)	26 (51.0%)		
Hostel	63 (28.4%)	159 (71.6%)		
Outside the college campus	17 (41.5%)	24 (58.5%)		
Smartphone addiction			27.506 (1)	<0.001^**^
Present	35 (20.6%)	135 (79.4%)		
Absent	70 (48.6%)	74 (51.4%)		

**Figure 2:**
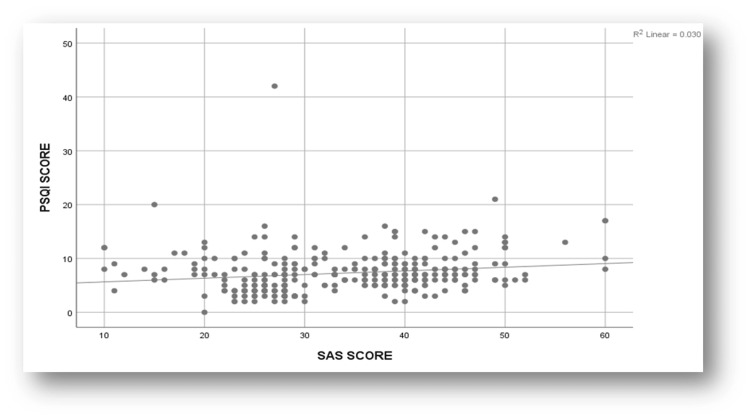
A scatterplot depicting correlation between Smartphone Addiction Scale-Short Version (SAS-SV) score and Pittsburgh Sleep Quality Index (PSQI) scores among medical students

Figure 2 illustrates the scatterplot of the correlation analysis between SAS-SV and PSQI Scores. The plot showed that there was a slightly positive correlation (r=0.172) between smartphone addiction scores and sleep scores, which was statistically significant (P<0.01).

## Discussion

The present study reported an overall smartphone addiction prevalence of 54.14% among the participants, which is higher than a similar study by Nowreen N and Ahad F in Jammu & Kashmir, India (34.4%) and the study conducted by Kurugodiyavar MD et al. in Karnataka, India (48.75%). In the neighboring country of Nepal, smartphone addiction was also high, present in more than two in five undergraduate students in this study [12-14]. It is now well documented that medical students extensively use mobile applications for a variety of educational purposes, including access to online textbooks, medical podcasts, calculators, recorded lectures, note-taking, and communication. The COVID-19 pandemic has further intensified this trend, as nowadays learning methods have shifted predominantly to virtual platforms.

As per the SAS-SV scale, the overall mean score was 34.01+9.78, which is higher than the previous Indian study (27.16) and another study conducted in Belgrade and Serbia, which reported the mean score as 24.69+9.14 [12,15]. Such variations in smartphone addiction rates among students across regions or countries may be attributed to differences in social and cultural contexts, as well as disparities in the development of and access to information and communication technologies.

Notably, male students (57.5%) were more prone (p < 0.05) to smartphone addiction than females (46.2%), which is in line with other studies in North India, where the prevalence of smartphone addiction was found to be 46.15% in males and 33.33% in females, and in Nepal (b = 5.3, p < 0.05) [14, 16]. However, in the study conducted in another country, no significant difference was observed between the prevalence amongst males and females [15].

Further analysis of smartphone usage revealed that the average daily screen time exceeded two hours, which is a matter of concern, as such prolonged use may negatively impact students’ academic responsibilities and time management. In the current study, 80.5% admitted to 'using smartphones longer than intended,' which is in agreement with another study conducted among nursing students in West Bengal, India, which reported that out of 91 students, 80 rated a score of 3 or higher on the Likert scale in response to the statement, 'I always think that I should shorten my smartphone-use time,' with 64 students selecting a score of 5 or 6 (i.e., agree or strongly agree). This indicates a high level of self-awareness among students regarding their excessive smartphone use and a willingness to reduce it [17].

According to the present study, the majority (66.6%) of medical students were poor sleepers (PSQI scores more than 5), which is in concordance with several other studies in India and abroad. In the study by Nowreen N and Ahad F., 62.7% of students had poor sleep quality, while in a study by Goel, A et al., approximately one in two respondents (48.3%) had a poor sleep index [12, 18]. Similar findings were reported in a study conducted among medical students in Cairo, where approximately two-thirds (63.1%) of the participants had a PSQI score greater than 5 [19].

As measured by the PSQI, the overall mean of sleep quality in our study was 7.28 (SD 3.86) among the medical students, which was higher than the previous study in Uttarakhand. India (5.03+0.86) [17]. Further, the components with the highest scores (indicating the worst sleep quality) in our study were sleep efficiency (2.26, SD 1.86) and sleep latency (1.24, SD 0.84), while the lowest scores were found in the use of sleep medication (0.22, SD 0.66) and subjective sleep quality (0.59, SD 0.73). A multicentric study across Latin America also revealed the same PSQI scores (7.26+3.45). However, the components with the highest scores were subjective sleep quality (1.33, SD 0.77) and daytime dysfunction (1.31, SD 0.84), while the lowest scores were found in the use of sleep medication (0.27, SD 0.70) and habitual sleep efficiency (0.89, SD 1.12), unlike our study [20]. Thus, poor sleep appears to be pervasive among medical students.

Interestingly, this study found that although smartphone addiction was predominant in male students, females were observed to have poorer sleep quality (71.0%). However, these findings contrast with the study by Kurugodiyavar MD et al. and Elsheikh AA et al., where males constituted 67% and 53% of poor sleepers, respectively, and this difference observed was statistically significant [13, 19]. Further in our study, as the age of students progressed (>24 years), they tended to suffer from poor sleep quality, although the association was insignificant (70.4%, p=0.865). This finding is in line with a previous study where there was no statistically significant association between age group or year of study with sleep quality [13].

Regarding other sociodemographic factors related to sleep quality, poor sleep quality was significantly associated with those students residing in the hostel (71.6%, p=0.01) and those having smartphone addiction (79.4%, p<0.001). Similar results were documented by previous studies where poor sleep was prevalent among those living in hostels (88.1%) [12, 19]. Poorer sleeping habits among hostel dwellers may be attributed to multiple factors, including peer pressure, increased stress levels, and homesickness.

The current study revealed a weakly positive correlation between the overall PSQI scores and SAS-SV scores, which was statistically significant (r=0.172, p<0.01). These are in accordance with multiple studies such as Kurugodiyavar MD et al. (r=0.343, p<0.001); Chatterjee S et al. and Goel A et al. (r = 0.182, p = 0.003) [13, 16, 18]. A systematic review and meta-analysis by Leow MQH et al. also stated that the overall correlation between SAS-SV and PSQI in their study was considerably low (r = 0.3). One possible explanation is that poor sleep quality is multifactorial and can be attributed to factors including academic stress, heavy study load, extended study hours, studying immediately before bedtime, irregular sleep-wake schedules, and anxiety related to academic performance and examination outcomes [21].

However, smartphone addiction appears to be a significant contributing factor adversely affecting sleep quality. Various mechanisms have been proposed to explain this relationship. Mobile phone addiction may impair sleep quality due to engaging in smartphone activities like gaming and social media, which often displace sleep. Features such as infinite scroll and notifications promote prolonged use, while blue light disrupts melatonin production and circadian rhythms, further exacerbating sleep disturbances. These effects are particularly concerning for medical students due to their irregular schedules and elevated stress levels [20].

Limitations of the study

Firstly, the single-time-point assessment limits the ability to infer a causal relationship between smartphone use and poor sleep quality. Secondly, as both sleep quality and the duration of smartphone use were self-reported by participants, the findings may be influenced by social desirability bias, where students might exaggerate their academic use or respond in a manner they believe is socially acceptable. Thirdly, the study included only medical undergraduates from a single institution, hence limiting the generalizability to other colleges, courses, or regions. Future studies should therefore incorporate more objective assessment methods alongside self-reported measures to facilitate a more accurate and nuanced evaluation of sleep patterns.

Recommendations

As future healthcare professionals, medical students must learn to use this double-edged sword judiciously rather than becoming slaves to modern inventions. Educational institutions should incorporate sleep education into medical curricula, emphasizing its critical role in cognitive performance and overall well-being. Furthermore, the promotion of behavioral interventions like scheduled 'digital detox' and the encouragement of digital tools that monitor and regulate screen time may assist students in cultivating healthier phone usage habits.

The study also advocates for enhancing self-awareness among students regarding help-seeking behaviors to manage smartphone use effectively. Providing counseling and support services during their formative academic years can be instrumental in fostering healthier habits. Tailored interventions aimed at stress management and effective time utilization may help address the underlying factors contributing to smartphone overuse and promote improved sleep hygiene. Furthermore, public health policymakers should consider such evidence when formulating guidelines on smartphone usage to advocate for healthier sleep behaviors at a broader level.

## Conclusions

The study revealed a high prevalence of smartphone addiction among medical students, with male students showing greater susceptibility. Given that adequate sleep is vital for cognitive performance, emotional stability, and overall health, these findings highlight an urgent need to address digital overuse in academic settings. Early interventions, such as digital wellness education, counseling support, and awareness programs promoting responsible smartphone use, can play a crucial role in mitigating this growing concern. Future longitudinal studies are warranted to explore the causal link between smartphone dependency and sleep disturbances and their broader implications on academic performance.

## Figures and Tables

**Figure 3:**
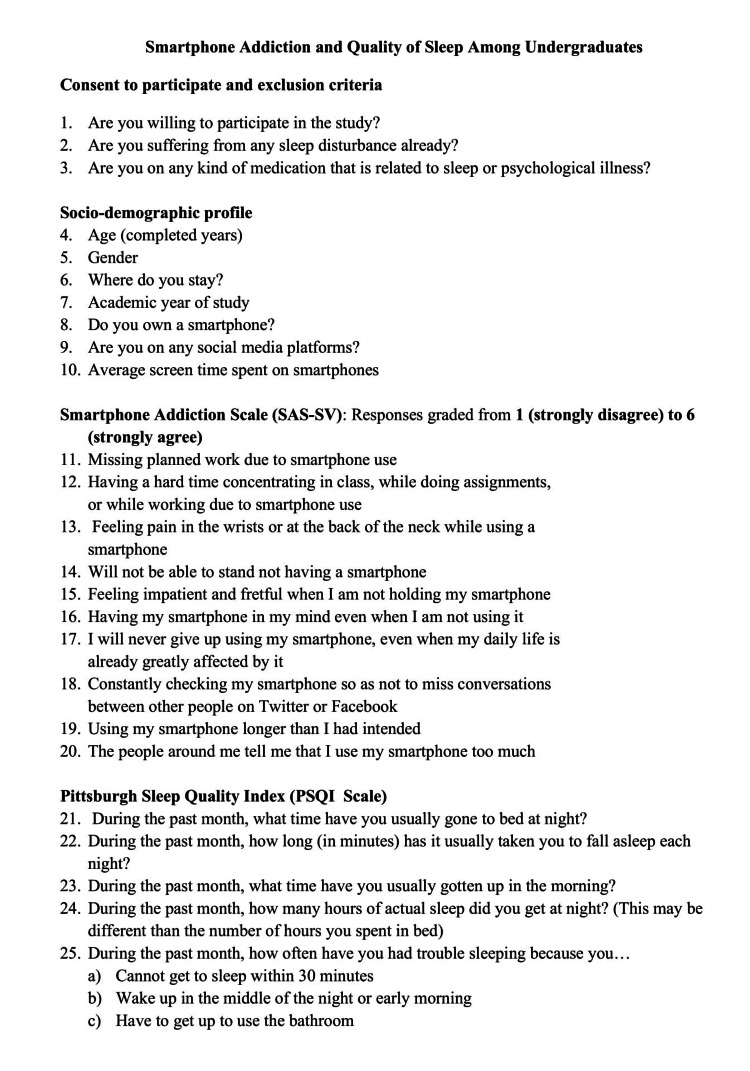
Research questionnaire part 1

**Figure 4:**
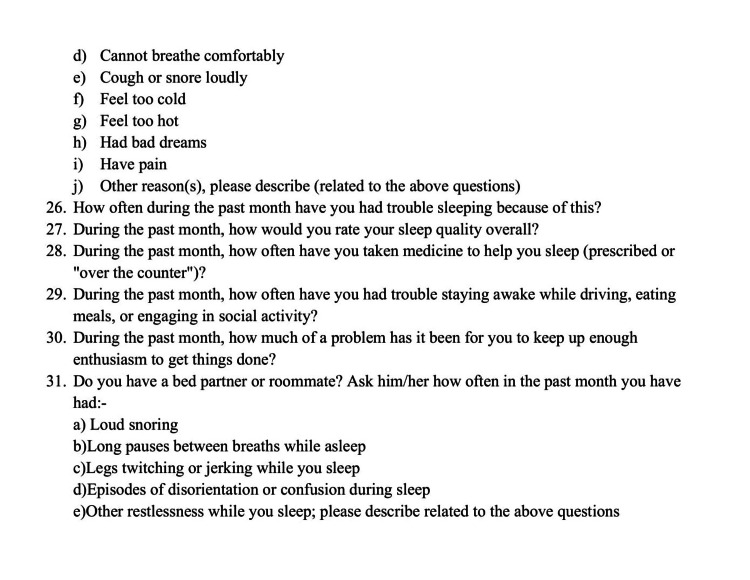
Research questionnaire part 2

## Appendices

Appendix A
